# Supplementary material for: Allosteric nanobodies uncover a role of hippocampal mGlu2 receptor homodimers in contextual fear consolidation
Source: Nat Commun. 2017 Dec 6;8:1967. doi: 10.1038/s41467-017-01489-1 (PMC5719040; doi:10.1038/s41467-017-01489-1)
Supplement: Supplementary file 1 — Supplementary Information [file 41467_2017_1489_MOESM1_ESM.pdf]

Nanobody	Conditions	Kd (nM) (mean ± SD)	n
DN1	Buffer	2.4 ± 0.5	12
	Agonist	2.1 ± 0.4	5
	Antagonist	2.5 ± 0.6	5
DN10	Buffer	NB	9
	Agonist	2.2 ± 0.4	10
	Antagonist	NB	7
DN13	Buffer	NB	9
	Agonist	3.5 ± 0.6	8
	Antagonist	NB	7

Supplementary Table 1. Apparent dissociation constant (Kd) of DN1, DN10 and DN13 measured using the TR-FRET binding assay as shown in Fig. 1, and control condition (buffer) or in the presence of the agonist LY379268 at saturating concentration (1 μM), or the antagonist LY341495 (10 μM). Values are mean ± SD of n independent determinations. NB, no binding.

Receptor	Mutant	Protomer A	Protomer B
mGlu2	A248K	-	A248K
	Mut A	L226Q, R445T, I450M	-
	Mut B	-	S246I, A248K, A249S, E251D, G252S
mGlu3	Mut A	Q232L, T458R, M463I	-
	Mut AB	Q232L, T458R, M463I	I252S, K254A, S255A, D257E, S258G

Supplementary Table 2. Description of the indicated mGlu2 mutants bearing mGlu3 specific residues from protomer A (Mut A) and protomer B (Mut B) and mGlu2 A248K mutant. Similar description for the indicated mGlu3 mutants.

Receptor	Mutant	Kd DN1 (nM)	Kd DN10 (nM)	Kd DN13 (nM)
mGlu2	WT	2.1 ± 0.4 (n=5)	2.2 ± 0.4 (n=10)	3.5 ± 0.6 (n=8)
	A248K	2.4 ± 0.3 (n=5)	5.3 ± 1.0 (n=7)	68.5 ± 2.7 (n=7)
	Mut A	2.4 ± 0.8 (n=6)	5.8 ± 3.2 (n=6)	1.6 ± 0.4 (n=6)
	Mut B	2.4 ± 0.9 (n=6)	12.4 ± 1.9 (n=8)	NB
mGlu3	WT	NB	NB	NB
	Mut A	NB	NB	NB
	Mut AB	NB	NB	29.5 ± 2.10 (n=6)

Supplementary Table 3. Apparent dissociation constant (Kd) of DN1, DN10 and DN13 measured as in Supp Table 1, for the indicated mutations described in Supp Table 2. Values are mean ± SD of n independent determinations as indicated. NB, no binding.

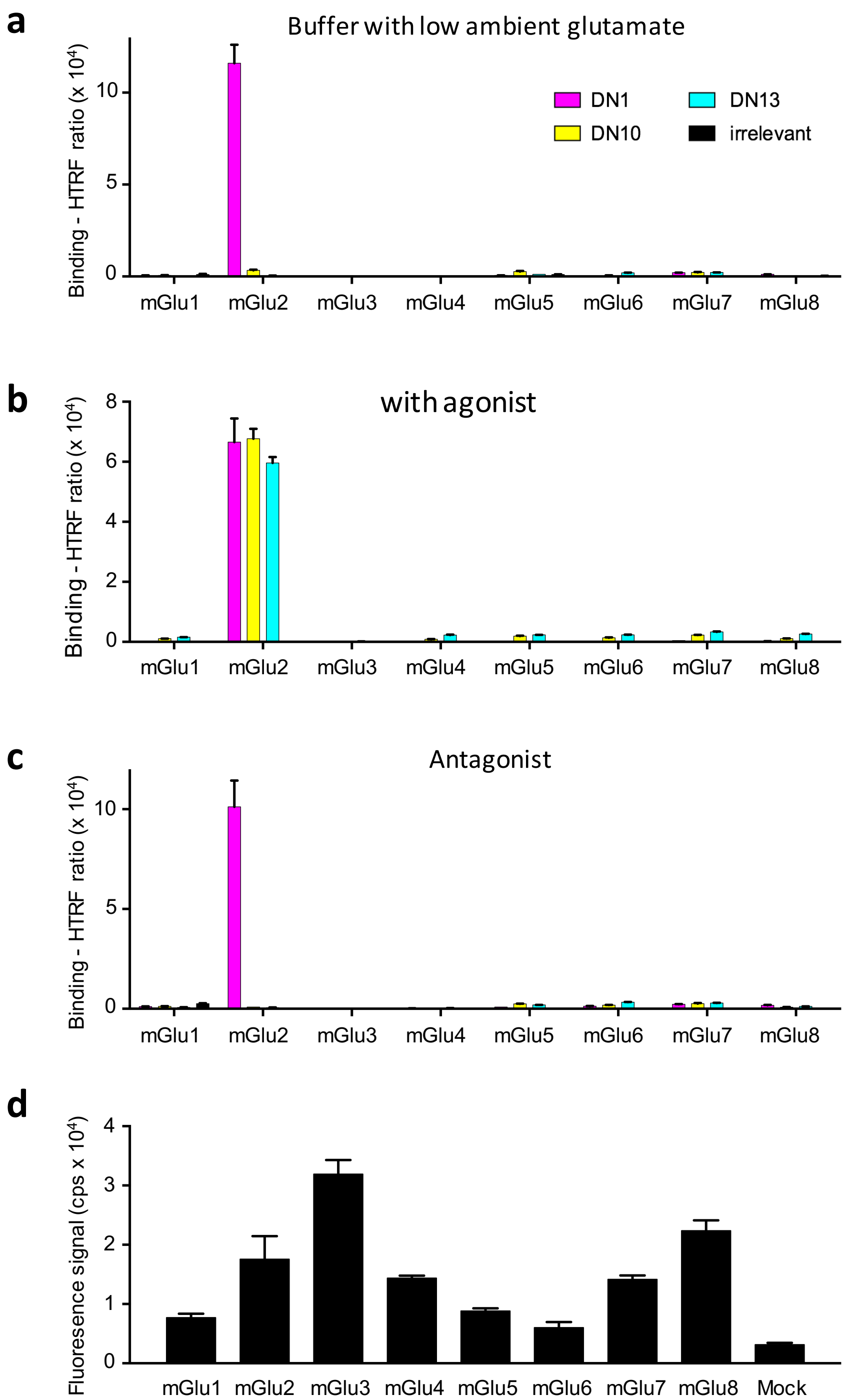

Supplementary Figure 1. Specific TR-FRET binding data obtained with the indicated SNAP-tagged mGlu receptor labeled with Lumi4-Tb and either DN1, DN10, DN13 or a control irrelevant nanobody detected with an anti c-Myc antibody carrying the d2 acceptor, in cells co-expressing the high affinity glutamate transporter EAAC1. **a)** Data obtained under basal condition, **b)** in the presence of agonist (quisqualate 100 µM for mGlu1 and 5; LY379268 for mGlu2 and 3; LSP4-2022 100 µM for mGlu4, 6, 7 and 8), **c)** or with antagonist (LY341495 10 µM for mGlu1 and 5; 5 µM for mGlu4 and 6; 0.5 µM for the other mGluRs). **d)** Expression levels of the various receptors at the cell surface as estimated by the Tb fluorescence intensity. Data are mean ± SD of triplicates from a typical experiment representative of three independent experiments.

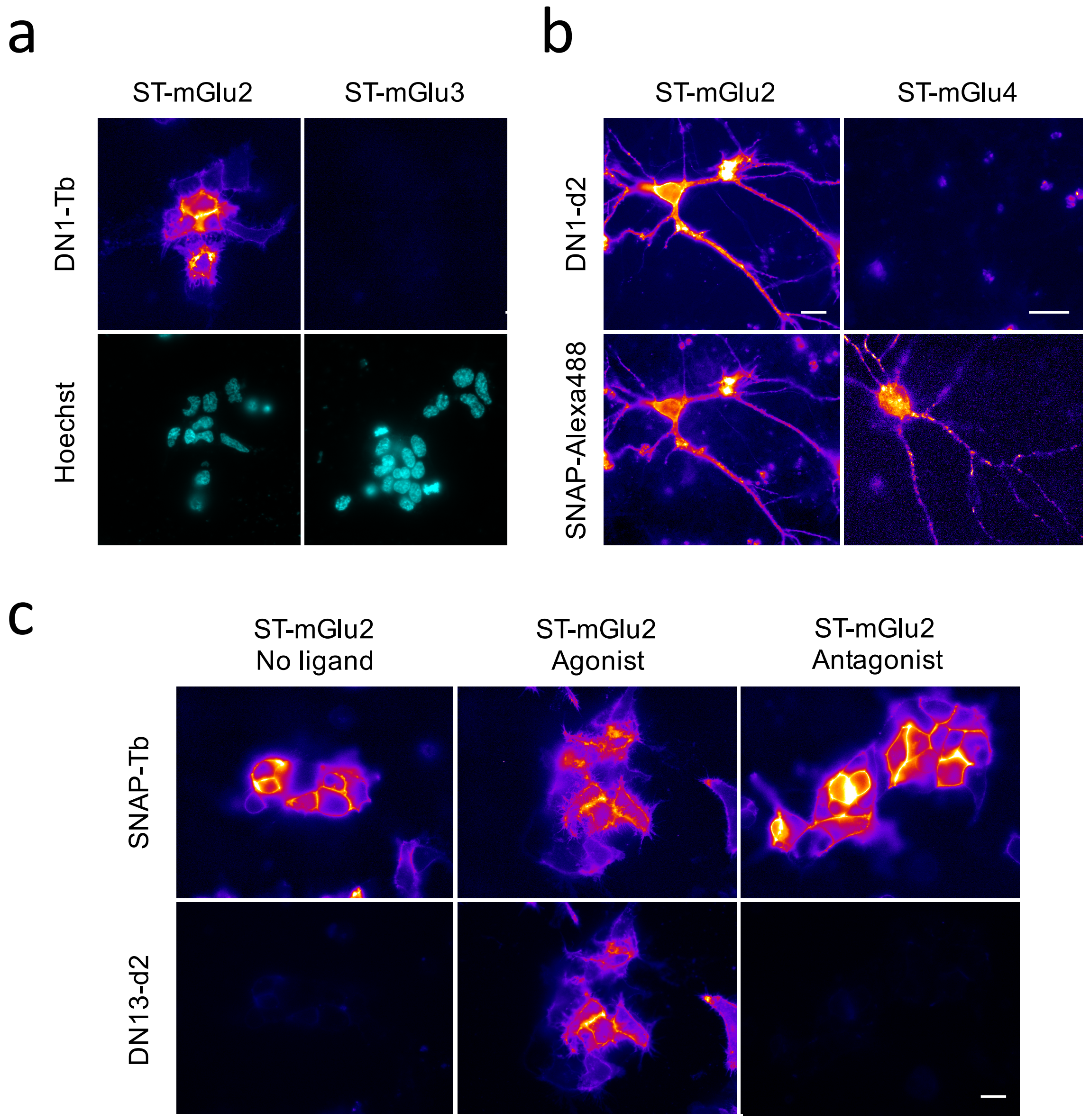

Supplementary Figure 2. Staining of transfected cells with fluorescent nanobodies. **a)** HEK-293 cells expressing either SNAP-mGlu2 (left) or SNAP-mGlu3 (right) receptors were labeled with DN1-Tb (top) or Hoechst 33342 to label nuclei (bottom). **b)** Cultured hippocampal neurons expressing either SNAP-mGlu2 (left) or SNAP-mGlu4 (right) receptors were labeled with DN1-d2 (top) or SNAP-Alexa488 (bottom). **c)** HEK-293 cells expressing SNAP-mGlu2 were labeled with SNAP-Tb (top) or DN13-d2 (bottom) under basal (left), agonist (LY379268 1 μM, middle), or antagonist (LY341495 10 μM, right) conditions. Scale bar: 20 μm.

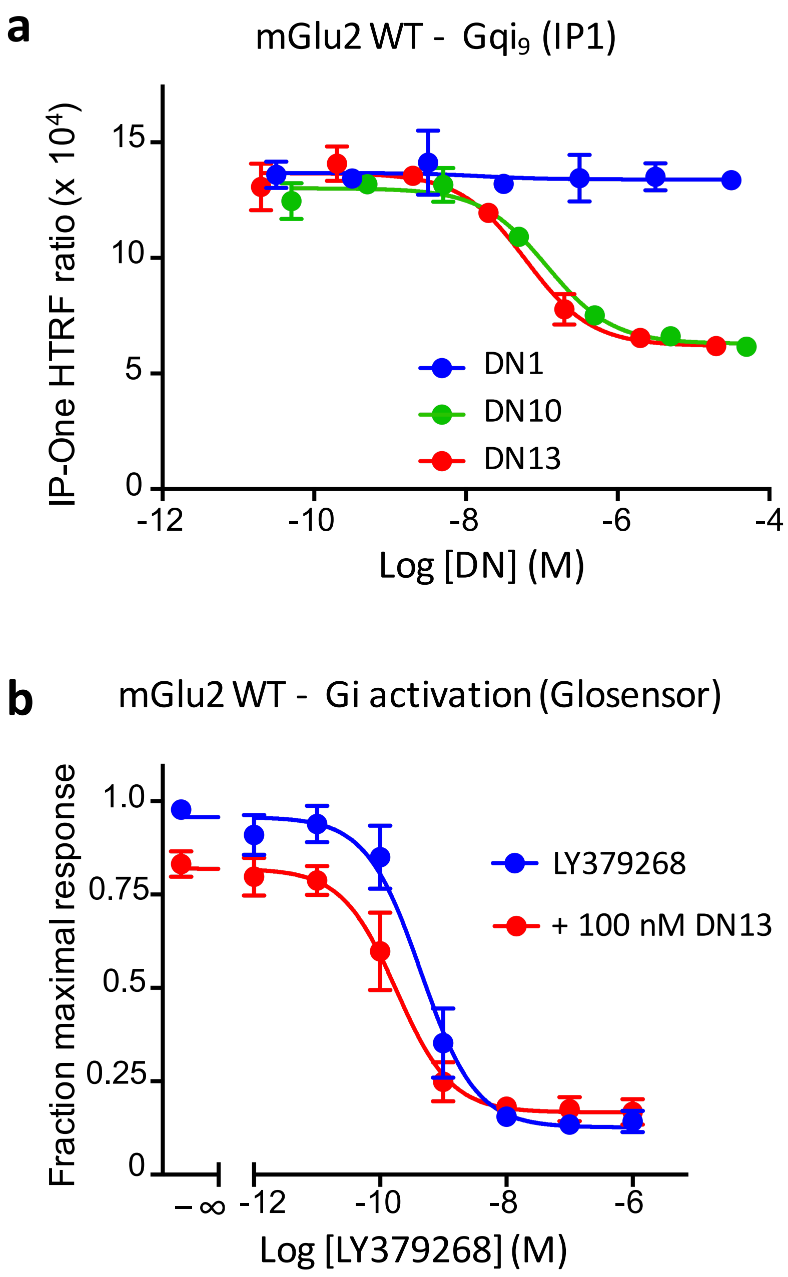

Supplementary Figure 3. Effect of nanobodies on the coupling of mGlu2 to Gqi9 and Gi proteins. **a)** IP1 production induced by increasing concentrations of DN1, DN10 and DN13 in cells expressing mGlu2 and the Gqi_9 protein in the presence of an EC20 of glutamate (1 µM). Data are expressed as the HTRF ratio of the IP-One assay kit. Note that the lower the HTRF ratio, the higher is the amount of IP1 in the assay. **b)** Inhibition of 1 µM forskolin-induced cAMP production by increasing concentrations the mGlu2 agonist LY379268 in absence (blue curve) and presence of 100 nM DN13. cAMP levels were measured by recording the luminescence signal of the pGlosensor. Data are mean ± SEM of three independent experiments performed in triplicates.

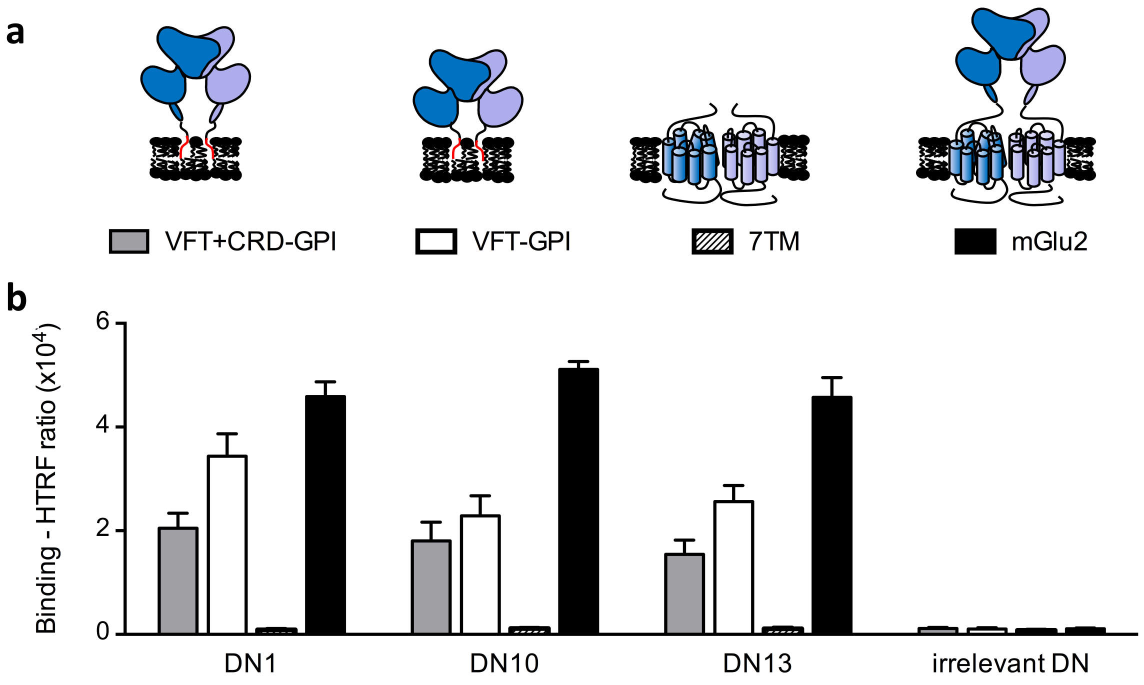

Supplementary Figure 4. DN1, DN10 and DN13 interact with the VFT of mGlu2 receptor. **a)** Cartoons representing the four different constructs tested. All are SNAP tagged at their N terminus. **b)** Binding of the indicated nanobodies on the constructs represented in a) (bar colors correspond to those indicated in a)) as quantified by TR-FRET according to Fig. 1a. Data are mean ± SD of triplicates from a typical experiment representative of three independent experiments.

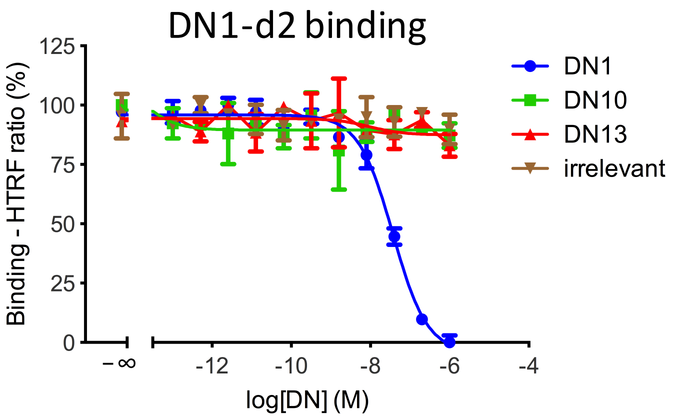

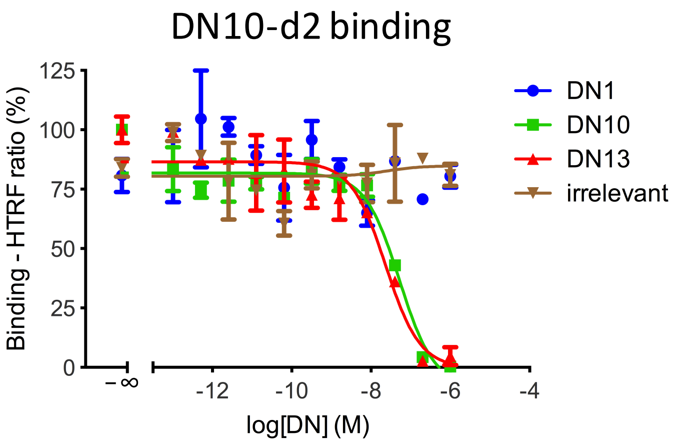

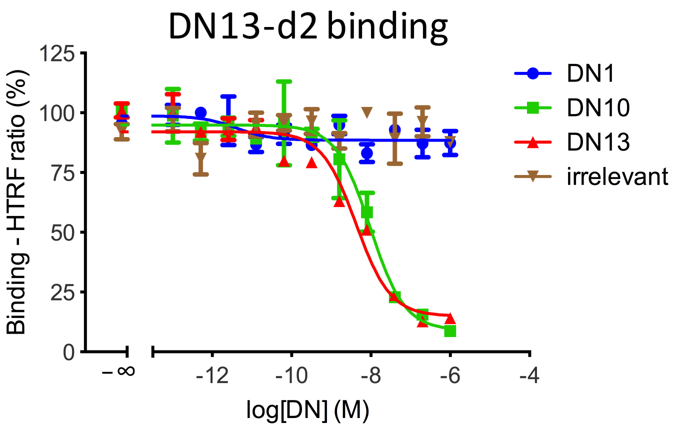

Supplementary Figure 5. DN10 and DN13 bind on overlapping epitopes, different from that recognized by DN1. The binding of the indicated d2-conjugated nanobody (DN1-d2 20 nM, DN10-d2 and DN13-d2 75 nM) to Lumi4-Tb labeled SNAP mGlu2 receptors was measured in the presence of increasing concentration of unlabeled DN1 (blue), DN10 (green), DN13 (red) or irrelevant nanobody (brown). According to the measured Kd of DN10-d2 (3.3 ± 0.5, n=3) and DN13-d2 (61 ± 14, n=5), and the means IC_{50} of the unlabeled nanobodies, Ki values for DN10 were 2.2 and 4.3 for inhibiting DN10-d2 and DN13-d2 binding respectively, and Ki values for DN13 were 1.9 and 1.0 against DN13-d2 and DN10-d2, respectively, consistent with a competitive inhibition. Data represented as percent of specific signal, and are mean ± SEM of three independent experiments performed in triplicates.

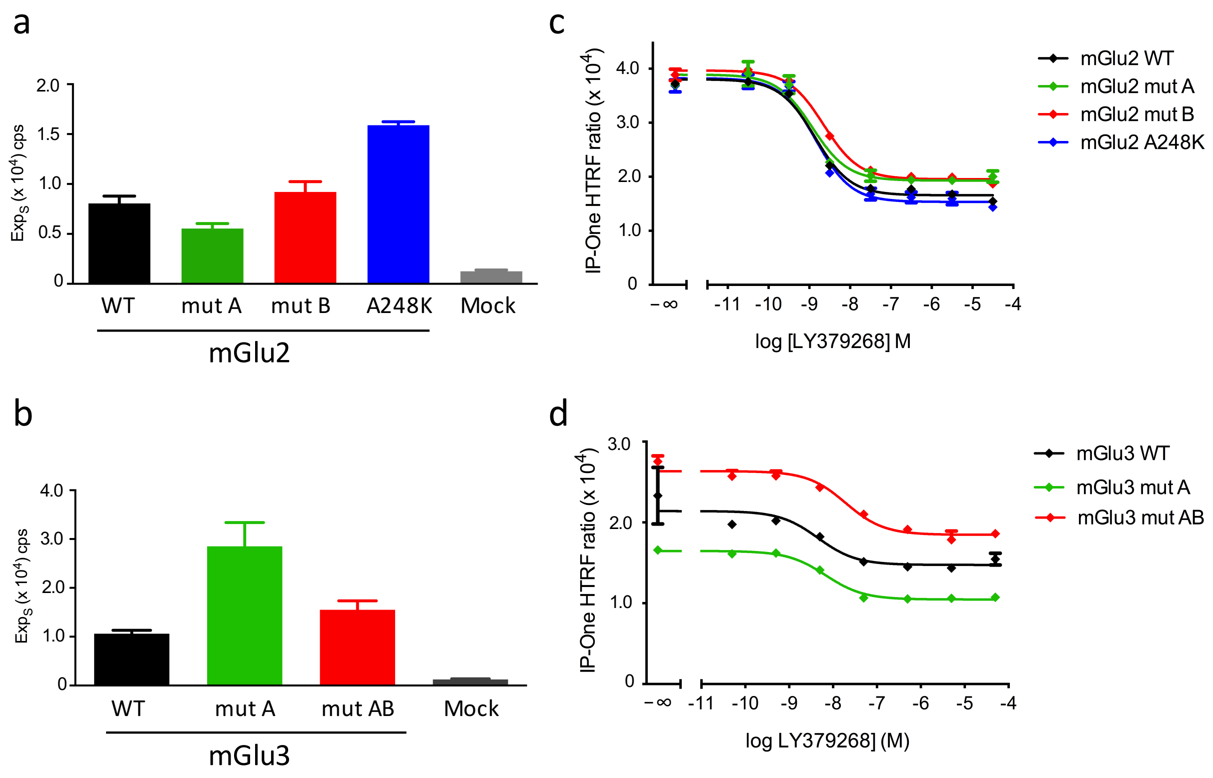

Supplementary Figure 6. mGlu2 and mGlu3 mutants are functional. Cell surface expression of the SNAP-tagged mGlu2 **(a)** and mGlu3 **(b)** mutants measured by the fluorescence emission of Lumi4-Tb attached to the SNAP-tag. IP1 accumulation for indicated mGlu2 **(c)** and mGlu3 **(d)** mutants stimulated by the agonist LY379268. Data represented as percent of specific signal, and are mean ± SEM of three independent experiments performed in triplicates.

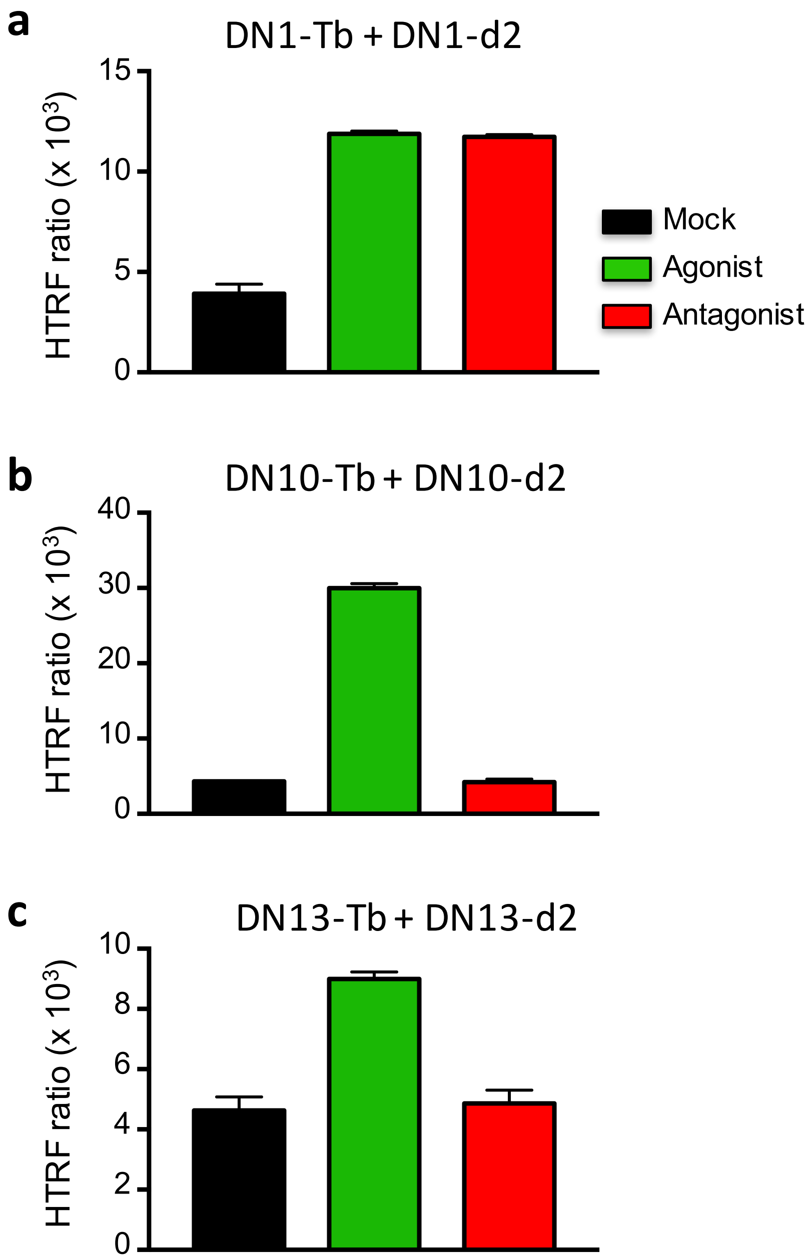

Supplementary Figure 7. Two nanobodies bind per mGlu2 homodimers. Mock transfected cells, or cells expressing mGlu2 receptors were incubated with d2-labeled and Lumi4-Tb-labeled nanobodies, and the TR-FRET signal was measured under control condition (Black column), in the presence of 10 µM LY379268 (agonist, Green column) or 10 µM LY341495 (antagonist, Red column). The concentrations of nanobodies used were: DN1-Tb 25 nM; DN1-d2 25 nM; DN10-Tb 10 nM; DN10-d2 10 nM; DN13-Tb 32 nM; DN13-d2 32 nM. Data are mean ± SEM of triplicate determinations from one experiment representative of 3 independent experiments.

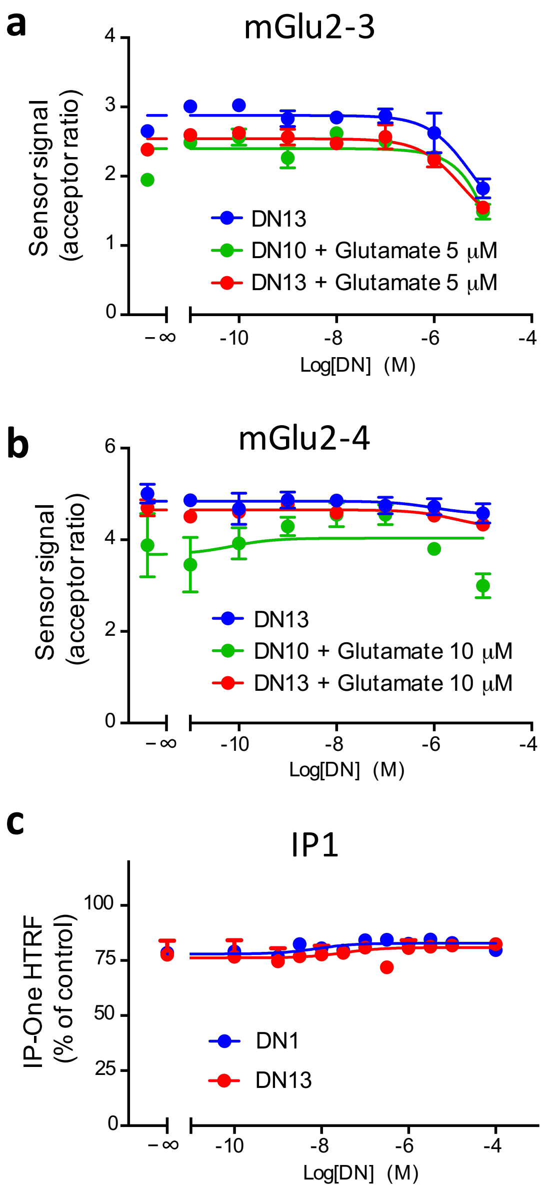

Supplementary Figure 8. Effect of DN10 and DN13 on the activity of mGlu2-3 and mGlu2-4 heterodimers. **a)** Effect of increasing concentrations of DN13 under control conditions (blue), and effect of DN10 and DN13 in the presence of 5 µM glutamate (green and red curves respectively) on the mGlu2-3 TR-FRET-based sensor. **b)** same as in a) with the mGlu2-4 sensor. In a) and b) TR-FRET signal was measured on cells expressing CLIP-mGlu2 and SNAP-mGlu3 or SNAP-mGlu4 labeled with CLIP-Lumi4-Tb and SNAP-d2. A decrease in TR-FRET signal is indicative of receptor activation. **c)** Effect of DN1 and DN13 on IP1 accumulation by mGlu2-4 heterodimer in the presence of 1 µM glutamate concentration. The heterodimer was composed of mGlu2-C1, a mGlu2 subunit carrying the C-terminal domain of $GABA_{B1}$, co-expressed with mGlu4-C2, a mGlu4 subunit carrying the C-terminal domain of $GABA_{B2}$ in which an endoplasmic retention motif has been added [1]. Note that under such conditions, neither mGlu2-C1, nor mGlu4-C2 reach the cell surface alone, or as homodimers. Only dimers composed of a C1 and a C2 containing subunits can reach the cell surface and be activated by mGluR agonists [2]. Data are mean ± SEM of three independent experiments performed in triplicates.

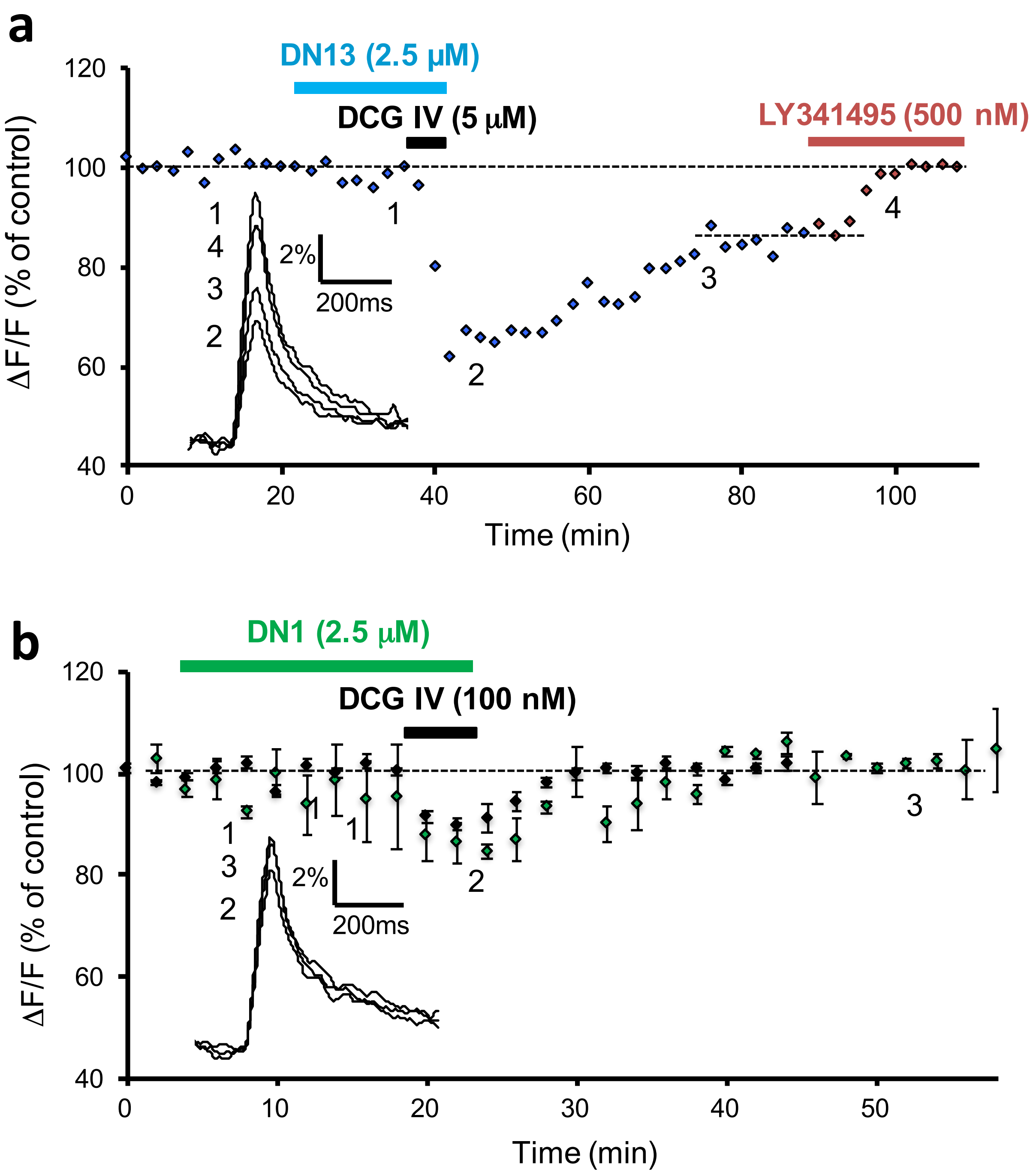

Supplementary Figure 9. Inhibition of the DN13 effect on the mossy fiber terminals by the mGlu2/3 antagonist, and absence of effect of DN1. **a)** Normalized amplitudes of peak fluorescence transients (ΔF/F) evoked in mossy fiber terminals generated by electrical stimulation (as depicted in Figure 5a, 5 stimulations at 100 Hz) are inhibited by a combination of both DN13 (2.5 μM) and DCG-IV (5 μM). This effect is inhibited by LY341495 (500 nM). **b)** Same experiment as in a) but with application of DN1 (2.5 μM) alone, and with DCG-IV at low concentration (100 nM). The lower right insets display superimposed averaged (10 sequential trials) fluorescence changes in one of these experiments recorded at the indicated times.

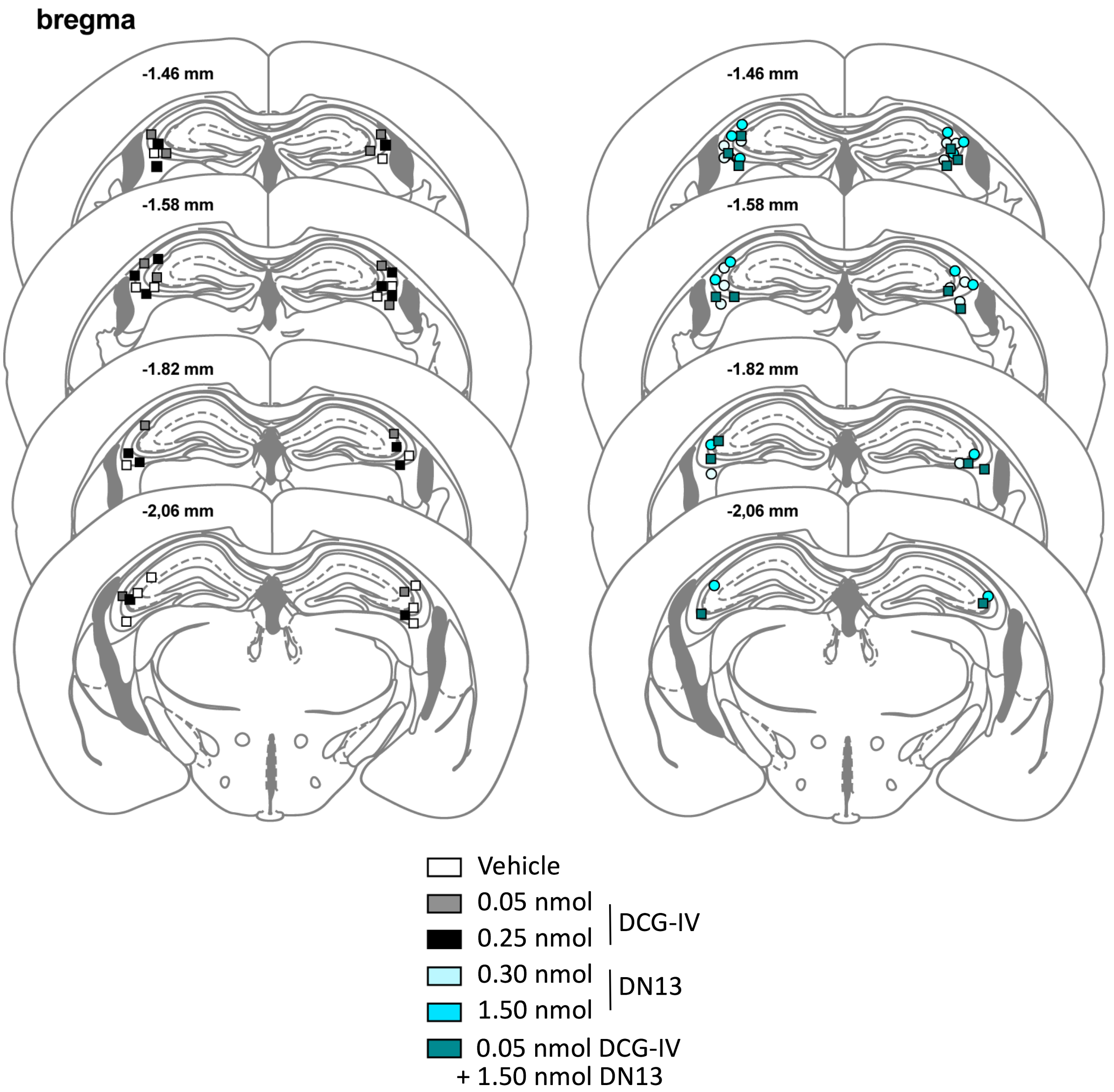

Supplementary Figure 10. Localization of the cannula, as revealed after slice preparation of the mouse brain used in the contextual fear conditioning test presented in Fig. 4e,f. Implantation points are color coded according to the group of animals.

References:

1. Scholler et al. HTS-compatible FRET-based conformational sensors clarify membrane receptor activation. Nat Chem Biol 13(4):372-380 (2017).
2. Moreno-Delgado et al. Pharmacological evidence for a metabotropic glutamate receptor heterodimer in neuronal cells. Elife 6, pii: e25233 (2017).
